# Chronic Pain and Chronic Stress: Two Sides of the Same Coin?

**DOI:** 10.1177/2470547017704763

**Published:** 2017-06-08

**Authors:** Chadi G Abdallah, Paul Geha

**Affiliations:** 1 Department of Psychiatry, Yale University School of Medicine, New Haven, CT, USA; 2 National Center for PTSD—Clinical Neurosciences Division, US Department of Veterans Affairs, West Haven, CT, USA; 3 The John B. Pierce Laboratory, New Haven, CT, USA

**Keywords:** pain, stress, limbic circuitry, hippocampus, amygdala, depression, posttraumatic stress disorder

## Abstract

Pain and stress share significant conceptual and physiological overlaps. Both phenomena challenge the body’s homeostasis and necessitate decision-making to help animals adapt to their environment. In addition, chronic stress and chronic pain share a common behavioral model of failure to extinguish negative memories. Yet, they also have discrepancies such that the final brain endophenotype of posttraumatic stress disorder, depression, and chronic pain appears to be different among the three conditions, and the role of the hypothalamic-pituitary-adrenal axis remains unclear in the physiology of pain. Persistence of either stress or pain is maladaptive and could lead to compromised well-being. In this brief review, we highlight the commonalities and differences between chronic stress and chronic pain, while focusing particularly on the central role of the limbic brain. We assess the current attempts in the field to conceptualize and understand chronic pain, within the context of knowledge gained from the stress literature. The limbic brain—including hippocampus, amygdala, and ventromedial prefrontal cortex—plays a critical role in learning. These brain areas integrate incoming nociceptive or stress signals with internal state, and generate learning signals necessary for decision-making. Therefore, the physiological and structural remodeling of this learning circuitry is observed in conditions such as chronic pain, depression, and posttraumatic stress disorder, and is also linked to the risk of onset of these conditions.

## Why Stress and Pain?

Stress-related psychiatric disorders, including depression and posttraumatic stress disorder (PTSD), are highly prevalent disabling illnesses with limited treatment options and poorly understood pathophysiology.^1^ Chronic pain is a widespread pathology afflicting 20%–30% of adults. Moreover, while treatment options are available, chronic pain continues to seriously affect the life quality of patients, and almost half of pain suffering individuals do not achieve adequate pain management.^2^ Better understanding of the overlapping and distinguishing features of chronic stress and pain could provide greater insight into the neurobiology of these processes, as well as contribute to rational drug development for these often comorbid conditions.^3^ In the current brief review, we describe the commonalities and differences of stress and pain, while primarily focusing on the maladaptive processes of chronic pain and chronic stress.

Pain and stress are two distinguished yet overlapping processes presenting multiple conceptual and physiological overlaps. Stress can be defined broadly as a process by which a challenging emotional or physiological event or series of events result in adaptive or maladaptive changes required to regain homeostasis and/or stability.^4^ Pain is the collection of emotional and sensory perceptions, as well as motor behaviors, resulting from the activation of the nociceptive pathways in response to harmful stimuli. The ability of the organism to adapt to stress or pain by regulating the internal milieu and maintaining stability is termed *allostasis.* Pain and stress are both adaptive in protecting the organism, for example, from physical injury or starvation. However, if either of the two processes becomes chronic, it can lead to long-term “maladaptive” changes in physiology and consequently behavior, resulting in suffering and compromised well-being.^5^ Taken together, these conceptualizations are clearly overlapping and offer an opportunity for theoretical and experimental exchanges between the two fields of study.

Researchers have adopted two, mutually non-exclusive, models linking pain and stress. The first model considers pain as one type of stress that adds strain on the organism. For example, chronic back pain (CBP) is conceptualized as a stress overload^6^ *resulting* in an increased risk for depression, alcohol abuse, or weight gain.^5,7,8^ In this model, chronic pain leads to “wear-and-tear”—also termed *allostatic overload*—in the body and brain “from chronic dysregulation (i.e., over-activity or inactivity) of physiological systems that are normally involved in adaptation to environmental challenge.”^9^ These wear-and-tear alterations result in compromised well-being, and/or social and occupational dysfunction. Persistent experience of pain (i.e., over-activity) can burden the brain and lead to deficits in decision-making.^10–12^ Conversely, fear of movement that would exacerbate pain could lead to a more sedentary lifestyle (i.e., inactivity) and weight gain. The second model depicts the cases in which wear-and-tear precipitates chronic pain. In this model, patients are faced with unpredictable stress that triggers pain—a migraine attack, for example—and leads to a vicious circle of “feed-forward” maladaptive physiological responses such as inflammation and brain damage and hence increased vulnerability to persistence of pain.^13^ The two models do not necessarily contradict each other, but rather borrow from the stress literature to provide either a causal conceptualization of the onset and persistence of chronic pain or of its long-term consequences. They also emphasize that stress and pain can be two nodes in a vicious circle of maladaptive responses to environmental challenges leading to compromised well-being.

In this review, we examine the important overlap between chronic pain and stress, while emphasizing differences between the two phenomena, which could have separate and even opposite neurobiological effects. We describe the commonalities and differences between chronic stress and chronic pain, with a special emphasis on the neurobiological underpinnings, where the brain limbic system^14^ stands as a central mediator of these two phenomena. We discuss in particular whether chronic pain can be considered under the larger process of stress or whether the two phenomena have different biological processes.

## Socioeconomic Factors in Stress and Chronic Pain

There is evidence that disparity in many dimensions of socio-economic status (SES) such as income, education, and occupation, account for a significant variance of medical morbidities and mortalities.^15,16^ Studies have found a so-called “gradient” between occupational hierarchy and health disparities in adults. People in the bottom of the gradient have worse morbidities and mortalities.^15^ These SES disparities can in turn translate at the individual level to environmental stressors leading to a vulnerability to depression, substance use disorders, and obesity among others.^4,17^ Furthermore, children growing up in poor communities are at an increased risk of exposure to crime, economic hardship, and pollution^18^; this in turn can lead to adverse behavioral (e.g., emotional dysregulation)^17,19^ and neurodevelopmental outcomes (e.g., psychopathology and brain changes).^20,21^ While the brain is believed to be at the center of this process, the direct path linking SES factors to neurobiological brain adaptive and maladaptive responses remains largely unknown,^9^ with pain and stress as putative contributing factors.

The link between SES factors and exposure to stress is evident, given the broad definition of stress. However, the relationship between SES and chronic pain is less discernable. In the British Birth Cohort Study, a 45-year longitudinal study, increased risk for reporting pain as adults was found in individuals from a lower SES, and in those who experienced adverse life events as children. However, the increased risk was partly explained by other current life factors.^22,23^ In patients followed through the emergency room after a major physical trauma, a higher educational level was the only social factor associated with persistent back pain. Income and employment status before the injury were not associated with persistent back pain after the trauma.^24^ Educational level was also a protective factor against frequent knee pain in a cohort of Swedish patients examined for knee osteoarthritis.^25^ These findings support the presence of a link between social stressors, lower educational level, and onset of pain diatheses. Nevertheless, a recent literature review found no relationship between SES characteristics and the frequency of seeking a medical consultation for back pain.^26^ In addition, two longitudinal studies found no significant correlations between chronic pain, socio-demographic factors, adverse life events, and “dysfunction of the stress system.”^27,28^ These studies underscore the complexity of the relationship between social factors and chronic pain, while challenging the common wisdom of a direct link between social stressors such as low SES and the onset of chronic pain.

## The Neurobiology of Stress and Pain

**Figure 1.**
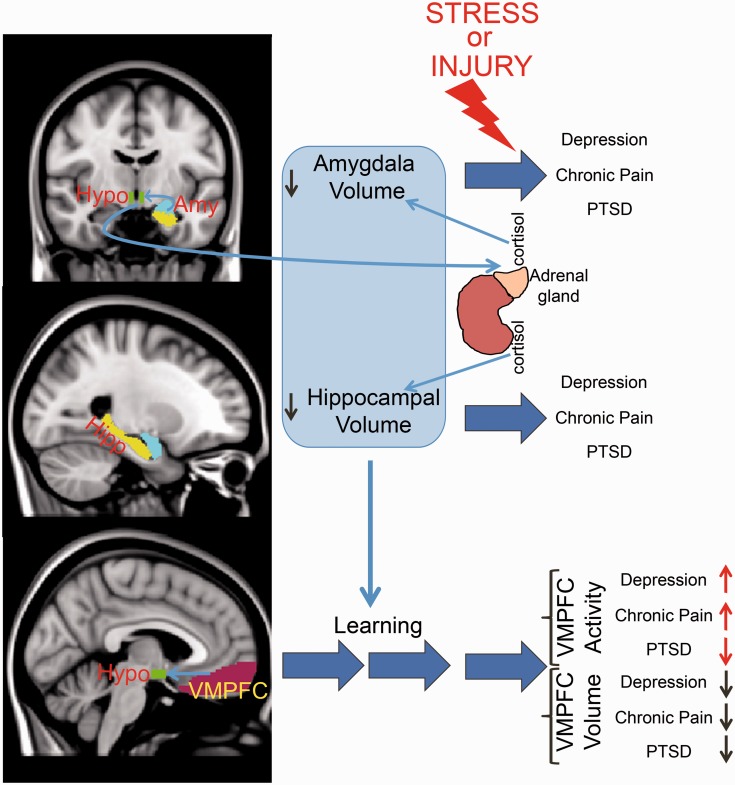
Schematic depiction of the circuitry involved in chronic pain and chronic stress. Light-blue arrows indicate anatomical or physiological links. Dark blue arrows indicate time. Black and red arrows indicate magnitude. Abbreviations: Amy, Amygdala; Hipp, Hippocampus; Hypo, Hypothalamus; PTSD, post-traumatic stress disorder; VMPFC, ventro-medial prefrontal cortex.

The brain plays a central role in stress and pain processes.^4,29,30^ As individuals interact with their environment, physical and psychological stressors can lead to adaptive or maladaptive neural and hormonal responses. Acute stress triggers the activation of the hypothalamic-pituitary-adrenal axis (HPA) leading to the release of adrenal glucocorticoids.^29^ These hormones have receptors concentrated in the limbic brain including the hypothalamus, amygdala, hippocampus, and prefrontal cortex (PFC).^31,32^ In the limbic system, glucocorticoids act as transcription factors and have therefore long-lasting effects on cellular function. Acute stress also activates the autonomic nervous system regulated by the brainstem,^33^ leading to increased blood pressure and diversion of blood from the gastrointestinal tract to the brain and muscles.^29^ In addition, perceived stress is integrated in the limbic brain with past experiences (i.e., memory), current physiological state (e.g., hunger/satiety), and decision-making. Subsequently, emotional states are updated accordingly (e.g., increased or decreased anxiety) with an ultimate effect on behavior (e.g., fight or flight). The limbic brain and HPA axis form an interconnected loop as projections from the hippocampus, amygdala, and PFC feed-back to the hypothalamus and regulate the stress responses and glucocorticoid release (Figure 1).^34^ Other brain areas have been also shown to be active during acute stress such as the insula and striatum.^35^

Pain requires conscious perception of the nociceptive process. Nociceptive information is transmitted via peripheral A-δ and C-fibers to the brainstem and thalamus, where it is then relayed to multiple cortical and subcortical areas including primary and secondary somatosensory areas, anterior cingulate cortex, insula, amygdala, striatum, and medial PFC.^36–39^ Acute pain engenders both a sensory and an emotional experience and is an adaptive response protecting the body from tissue damage like a burning fire or the attack of a predator. Although acute pain can be easily conceptualized as a form of acute stress, the details of the neural and endocrine response to acute pain and acute stress can be different. For example, while it is known that both acute pain and stress activate the autonomic nervous system,^29,40^ evidence that acute pain activates the HPA axis and leads to peripheral adrenal cortisol secretion, one of the hallmark endocrine responses to stress, is unclear.^41,42^ Alternatively, at the brain level, functional magnetic resonance imaging studies of response to stress or pain demonstrate noticeable spatial overlap in the amygdala, hippocampus, striatum, insula, and anterior cingulate cortex.^35,43^

## Learning and Neural Remodeling in Chronic Stress and Chronic Pain

Stress and pain engage the learning circuitry of the hippocampus, amygdala, and PFC (Figure 1), as animals interact adaptively with the challenges of their environment to maintain homeostasis.^9,43^ Animals have to learn about their environment to seek places where food can be found while avoiding the threat of an attack from a predator or the ingestion of poisonous substances. Fear (Pavlovian) conditioning and extinction are paradigms of learning in which both chronic pain and chronic stress can be conceptualized.^44,45^ In a Pavlovian model, a previously neutral stimulus acquires the ability to induce fear behavior in animals (e.g., conditioned stimulus) after being paired with an unconditioned painful stimulus, like a foot-shock.^46^ Extinction of the fear association, or the unlearning of the fear, occurs when the conditioned stimulus is presented multiple times without the unconditioned stimulus.^47^ PTSD and chronic pain can be considered conditions where the brain fails to extinguish the negative memory (i.e., memory of trauma or pain).^44,45^ Consistently, both PTSD and chronic pain patients show deficiency in extinction learning.^48,49^ In addition, similar to findings in traumatic stress preclinical literature,^50^ an animal model of chronic neuropathic pain shows impaired context-related fear extinction.^51^ The hippocampus, amygdala, and PFC each play a critical role in fear learning and extinction.^45,52,53^ The neurochemical properties of the learning circuitry and its adaptive response to chronic stress or pain are believed to be crucial in determining remission or persistence of pain and stress response beyond what is required for an evolutionary advantageous adaptive response.^45,54–56^ Below, we expand on details of the role played by each of these regions in chronic stress and chronic pain demonstrating the compelling conceptual overlap between the fields yet highlighting important empirical differences.

The hippocampus is active during acute stress,^35^ but rarely seen active during acute pain in humans.^57^ An intact hippocampus is important during acquisition of fear conditioning and association of context with stimuli that necessitates decision-making such as finding food or avoiding pain.^58–62^ In addition, the hippocampus contributes to contextual fear extinction.^63,64^ It contains glucocorticoid receptors,^65^ projects to the hypothalamus,^66^ and is thought to down-regulate the response to stress.^9,67–69^ Neurogenesis persists in the adult mammalian hippocampus^70^ and contributes to learning and memory.^71^ In humans, chronic pain and stress-related psychiatric disorders have been associated with shrinkage of the hippocampal volume.^51,56,72,73^ Vachon-Presseau et al. demonstrated that hippocampal volume is inversely correlated to elevated basal cortisol levels in CBP patients but not in matched healthy control arguing for a “stress model of chronic pain” centered on the hippocampus. Interestingly, smaller hippocampal volume predicts the risk of persistence of back pain after three years of a new episode of sub-acute back pain (SBP; pain duration 6–16 weeks),^56^ and is present in individuals at risk for PTSD and depression.^74,75^

Both chronic pain and stress were associated with suppressed hippocampal neurogenesis,^51,72^ a process that could be mediated by elevated glucocorticoids during stress.^72^ However, the relationship between neurogenesis and acute pain or stress is more complex. A recent study in rodents found that adult hippocampal neurogenesis is *necessary* for the emergence of pain behavior after nerve injury.^76^ Nevertheless, neurogenesis was suppressed once the pain became chronic,^51^ implying that the interaction between peripheral injury and central hippocampal learning mechanisms is critical for the onset of pain behavior. These results are in resemblance to findings by Kirby et al.^77^ showing that acute immobilization for 3 h, but not foot shock for 30 min, *increased* hippocampal neurogenesis. The effect of immobilization stress on neurogenesis could be reproduced with corticosterone injections, which leads to a delayed onset (at 2 weeks) of *enhanced* fear extinction.^77^ These studies highlight the fact that acute pain (i.e., foot shock) cannot be fully conceptualized as an acute stressor and that learning after nerve injury might have different long-term behavioral effects from stress. It also underscores the beneficial effects of acute stress, which increased neurogenesis and enhanced extinction. Consistently, Mutso et al.^51^ demonstrated *impaired* contextual fear extinction after peripheral nerve injury. In contrast, Kirby et al.^77^ demonstrated *enhanced* fear extinction two weeks after an acute stressor presentation.

The amygdala is another major node of the limbic brain (Figure 1) that is highly interconnected with the hippocampus.^78^ It plays a major role in emotional learning^53^ and in the response to stress and pain. The amygdala is active during response to threats such as angry faces^79,80^ and in response to acute pain.^81,82^ It is critical in the expression of fear^46^ and shows hyperactivity in chronic stress-related conditions such as PTSD, and in chronic pain disorders such as CBP or migraine.^83–85^ Animal data show that the amygdala plays a dual role in the perception of nociceptive input depending on the context of the painful stimulation. Lesion of the central nucleus of the amygdala (CeA) abolishes or decreases aversive stimulus-induced hypoalgesia (i.e., pain reduction).^86^ Corticosterone implant in the CeA enhances anxiety-like behavior and visceral hypersensitivity to balloon distention of the colon or acetic acid infusion in the colon.^87^ In addition, CeA neurons show increased sensitization in a rodent model of arthritis, independent of peripheral nociceptive input.^88^ In animal models, chronic stress and chronic pain are both associated with dendritic growth in the amygdala^89–91^ suggesting enhanced synaptic activity, possibly in response to increased glucocorticoid levels.^92^ At the macroscopic level, humans suffering from depression, PTSD, or chronic pain were found to have smaller amygdala,^56,93–96^ although not without inconsistency (e.g., Kuo et al.^97^). Interestingly, depressed patients on medications have increased amygdala volume.^93^ In addition, a cohort of 10 patients with hip osteoarthritis showed an increase in amygdala volume after total hip replacement and remission of pain^98^ suggesting that volume shrinkage is a consequence of chronic pain and depression, and could therefore recover if both conditions are adequately treated. This data, along with the decreased hippocampal volume in chronic pain, is consistent with the concept of *allostatic-load* from chronic pain as volumetric shrinkage can be considered the *wear-and-tear* manifested in the brain secondary to the chronic exposure to nociception lending support to the view that chronic pain can be considered a form of chronic stress.^6^ Nevertheless, other data showed that amygdala volume stays unchanged and predicts the persistence of back pain three years after a sub-acute episode of back pain,^56^ suggesting that a smaller amygdala volume could be a risk factor for chronic pain and not the consequence of exposure to chronic pain.

Both the hippocampus and amygdala are highly interconnected with the ventro-medial PFC (vmPFC)^99,100^ (Figure 1), which is a critical area in fear extinction^52,54,101,102^ and in assigning value to rewarding and aversive stimuli.^103,104^ vmPFC volume shrinks in chronic pain, PTSD, and depression.^105,106^ Activity in the vmPFC, on the other hand, increases after repeated acute stress in healthy subjects,^35^ and is increased in patients suffering from chronic pain and depression^84,85,107–109^ but is decreased in patients suffering from PTSD.^45,110^ In addition, vmPFC activity is positively correlated with pain intensity in CBP patients,^84,85,107^ but negatively correlated with severity of symptoms in PTSD. Therefore, the physiology of chronic pain and chronic stress might be diverging in the vmPFC. Behaviorally, altered vmPFC activity could explain impaired extinction in PTSD and impaired emotional decision-making in chronic pain^10,11,111,112^ and depression.^113^

## Contentious Points in Borrowing From Stress to Explain Chronic Pain

Despite the significant neuroanatomical and physiological overlap reported above between chronic pain and chronic stress, upholding the stress model of chronic pain faces some challenges. First, as we outlined above, the data on the contribution of psycho-social factors and markers of biological stress to the onset or the persistence of chronic pain is conflicting.^22,26–28^ Second, the data on the “dysregulation of the HPA axis and cortisol” level in chronic pain does not fit any clear consistent pattern. As such, studies of chronic pain conditions have reported increases^6,114–119^ and decreases^41,120–124^ in cortisol level, while many studies reported no changes.^125–128^ Furthermore, different reports present conflicting data within the same condition such as CBP,^6,41^ fibromyalgia,^125,129^ or migraine.^117,119,128^ Third, the definition of stress is very broad; for example, showing violent pictures and acute aversive stimuli-like acute pain can be both stressful but involve different physiology. Furthermore, release of cortisol and activation of the hippocampus are often observed following stress,^35^ but rarely seen after acute pain.^57^ Similarly, although chronic conditions that are thought to arise after repeated stress or trauma such as PTSD and depression share markers of vulnerabilities with chronic pain within the limbic brain like a smaller hippocampus and a smaller amygdala,^56,75,93–95^ the brain endophenotypes appear to be different. For example, vmPFC global brain connectivity is decreased in depression,^130^ yet increased in PTSD^131,132^ and chronic pain.^133^ This observation does not preclude a role of stress physiology in the onset and persistence of chronic pain, but rather calls for more specific definitions of the biological markers of stress.

## Conclusion

Taken together, the data discussed above provide a rationale for the attempts to use the stress model in chronic pain, yet emphasize the difficulties in classifying the concept of chronic pain under the general framework of chronic stress. We believe that unifying both processes under one theoretical framework would be enhanced by understanding how different chronic painful or stressful conditions induce continuous emotional learning centered particularly around the properties and remodeling of amygdala and hippocampus.
